# VirusHound-I: prediction of viral proteins involved in the evasion of host adaptive immune response using the random forest algorithm and generative adversarial network for data augmentation

**DOI:** 10.1093/bib/bbad434

**Published:** 2023-11-29

**Authors:** Jorge F Beltrán, Lisandra Herrera Belén, Jorge G Farias, Mauricio Zamorano, Nicolás Lefin, Javiera Miranda, Fernanda Parraguez-Contreras

**Affiliations:** Department of Chemical Engineering, Faculty of Engineering and Science, Universidad de La Frontera, Ave. Francisco Salazar 01145, Temuco, Chile; Departamento de Ciencias Básicas, Facultad de Ciencias, Universidad Santo Tomas, Chile; Department of Chemical Engineering, Faculty of Engineering and Science, Universidad de La Frontera, Ave. Francisco Salazar 01145, Temuco, Chile; Department of Chemical Engineering, Faculty of Engineering and Science, Universidad de La Frontera, Ave. Francisco Salazar 01145, Temuco, Chile; Department of Chemical Engineering, Faculty of Engineering and Science, Universidad de La Frontera, Ave. Francisco Salazar 01145, Temuco, Chile; Department of Chemical Engineering, Faculty of Engineering and Science, Universidad de La Frontera, Ave. Francisco Salazar 01145, Temuco, Chile; Department of Chemical Engineering, Faculty of Engineering and Science, Universidad de La Frontera, Ave. Francisco Salazar 01145, Temuco, Chile

**Keywords:** virus, pathogen, machine learning, neural network, deep learning, protein

## Abstract

Throughout evolution, pathogenic viruses have developed different strategies to evade the response of the adaptive immune system. To carry out successful replication, some pathogenic viruses encode different proteins that manipulate the molecular mechanisms of host cells. Currently, there are different bioinformatics tools for virus research; however, none of them focus on predicting viral proteins that evade the adaptive system. In this work, we have developed a novel tool based on machine and deep learning for predicting this type of viral protein named VirusHound-I. This tool is based on a model developed with the multilayer perceptron algorithm using the dipeptide composition molecular descriptor. In this study, we have also demonstrated the robustness of our strategy for data augmentation of the positive dataset based on generative antagonistic networks. During the 10-fold cross-validation step in the training dataset, the predictive model showed 0.947 accuracy, 0.994 precision, 0.943 F1 score, 0.995 specificity, 0.896 sensitivity, 0.894 kappa, 0.898 Matthew’s correlation coefficient and 0.989 AUC. On the other hand, during the testing step, the model showed 0.964 accuracy, 1.0 precision, 0.967 F1 score, 1.0 specificity, 0.936 sensitivity, 0.929 kappa, 0.931 Matthew’s correlation coefficient and 1.0 AUC. Taking this model into account, we have developed a tool called VirusHound-I that makes it possible to predict viral proteins that evade the host’s adaptive immune system. We believe that VirusHound-I can be very useful in accelerating studies on the molecular mechanisms of evasion of pathogenic viruses, as well as in the discovery of therapeutic targets.

## INTRODUCTION

There have been significant pandemics in the last century, such as the 1918 H1N1 influenza and HIV, which caused millions of deaths. In the twenty-first century, there have been zoonotic outbreaks, including SARS, MERS, Ebola, Hendra, Nipah and COVID-19, which have become a major crisis with global consequences [1]. Pathogenic viruses have evolved various strategies to evade the immune system and successfully infect host cells [2, 3]. The adaptive immune response is a key component of the host’s immune system against viral pathogens, and many of these pathogens have evolved to evade it [4]. Among the most common mechanisms used by pathogenic viruses to evade this type of response are inhibition of host major histocompatibility complex class I (MHC-I) molecule presentation [5–7], MHC class II (MHC-II) molecule presentation, proteasome antigen processing, transporter associated with antigenic processing (TAP), and tapasin [7]. For example, the human papillomavirus (HPV) encodes a protein called E5 (HPV E5) that facilitates successful infection by inducing loss of surface MHC-I expression in infected basal cells, thereby preventing viral antigen presentation to effector T cells. HPV16 E5 can bind to the transmembrane domain of MHC-I, retaining it inside the Golgi apparatus, thus preventing its trafficking to the cell surface [8, 9]. The US2 protein of human cytomegalovirus inhibits the MHC-II antigen presentation pathway by degrading human leukocyte antigen (HLA)-DR-α and -DM-α, thereby preventing recognition by CD4^+^ T cells [10]. Finally, the Epstein–Barr virus encoded nuclear antigen 1 protein interrupts proteasome substrate processing [11], among many other examples. The study of virus proteins that evade adaptive immune responses is crucial in the development of vaccines and therapeutic drugs that help combat these pathogens more efficiently [12]. However, despite advances in understanding the molecular mechanisms by which viruses evade the immune system, demonstrating that a virus protein confers the ability to evade the immune system remains a difficult and resource-intensive task. In this direction, the development of alternative tools that allow for an accelerated process is an area of research that must be considered to combat these unpredictable and dangerous pathogens.

In recent years, different immunoinformatic tools based on machine learning algorithms have been developed to address various problems in the field of immunology, which have been trained with experimental datasets of immunogenic proteins and peptides. Most of these tools focus on predicting the binding affinity of peptides to MHC-I and MHC-II, as well as predicting B-cell epitopes, based solely on the primary sequences of proteins [13, 14], which facilitates the large-scale study of potentially immunogenic peptides. On the other hand, there are other immunoinformatic tools for the study of viruses based on machine learning such as VirVACPRED and VaxiJen, which allow for specific predictions of viral antigens [15, 16]. Other alternatives for the study of viruses are tools for predicting the subcellular localization of these pathogens such as MSLVP [17], Virus-mPLoc [18] and pLoc_Deep-mVirus [19], among many other tools compiled in an excellent review by Kumar and colleagues [20]. To date, there is no tool for predicting virus proteins that evade the host adaptive immune response. In this direction, the present work aims to develop a novel tool to address this important problem. This tool could be of great use in the study of the molecular mechanisms by which these pathogens camouflage themselves against this type of response, as well as facilitating the discovery of new therapeutic targets.

## MATERIAL AND METHODS

### Datasets

In this study, we identified 98 virus proteins involved in evading the host adaptive immune response (VPEs) from the scientific literature. The primary reference sequences of these proteins were identified and downloaded from the UniProt database [21] to construct a positive dataset (positive dataset). On the other hands, to create the negative dataset, we randomly selected 285 virus proteins (negative dataset) lacking this biological functionality (non-VPEs) in the UniProt database. These were also compared with the scientific literature to ensure that they are not involved in invading the host adaptive immune response.

### Feature computation

From both datasets, four different types of molecular descriptors were calculated: amino acid composition (AAC, 20 features), amphiphilic pseudo amino acid composition (APAAC, 50 features), dipeptide composition (DPC, 400 features) and pseudo amino acid composition (PAAC, 50 features). All calculations of the molecular descriptors were performed using the propy3 package (https://propy3.readthedocs.io/).

### Data augmentation using generative adversarial network

#### GAN architecture

Considering the disproportion between the positive and negative datasets in terms of the number of virus protein sequences, we proceeded to balance this imbalance by generating synthetic data from the positive dataset. For this purpose, a Generative Adversarial Network (GAN) was used, and it was separately fed with each of the molecular descriptors calculated from the positive dataset. The implementation of our strategy for generating synthetic data consists of three models: the generator, the discriminator and the GAN. The generator was provided with a noise input, and synthetic data resembling the real data were generated from this input. Subsequently, the architecture was configured so that the discriminator received real or synthetic data and attempted to classify them as true or false. The GAN developed in this work combines the generator and the discriminator in a neural network to train them together. The generator seeks to improve its ability to generate increasingly realistic synthetic data, while the discriminator aims to improve its ability to distinguish between real and synthetic data. The GAN was trained in a zero-sum game process where the generator tries to deceive the discriminator, and the discriminator tries to correctly identify the fake data. This feedback process was iteratively repeated until the GAN was capable of generating synthetic data that is indistinguishable from the real data.

#### Generator configuration

A neural network was configured consisting of three central layers. First, an input layer with a dimensionality of 128 was used. Subsequently, hidden layers were implemented, each composed of two dense layers with 128 units. These layers are followed by LeakyReLU activations with a slope factor of 0.01 and dropout layers with a retention rate of 50%. Finally, the output layer was configured as a dense layer designed to generate synthetic data, maintaining the same dimensionality as the input dataset.

#### Discriminator configuration

An input layer with a dimensionality equal to the number of entities in the dataset was used. The hidden layers consist of two dense layers, each comprising 128 units. These layers were followed by LeakyReLU activations with a slope factor of 0.01 and dropout layers with a retention factor of 0.5. The output layer was configured as a dense layer with a sigmoid activation for binary classification of data as real or synthetic. The discriminator was trained to distinguish between real and synthetic data using the binary cross-entropy loss function and was optimized with the Adam algorithm, configured with a learning rate of 0.0002 and a beta factor of 0.5. Simultaneously, the GAN was trained over 1000 epochs, with a batch size of 30 examples per epoch, aiming to deceive the discriminator by generating synthetic data resembling real data. Losses for both the discriminator and the generator were recorded in each epoch, and these losses were summed as the evaluation metric.

The entire strategy carried out for generating synthetic data was written using the TensorFlow 2.0 framework (https://www.tensorflow.org/). The code written for this task was deposited along with all the code used in this work and the datasets in the GitHub repository: https://github.com/jfbldevs/virushound-I.

### Classification and assessments

The random forest classification algorithm (RF) was used to develop predictive models for viral proteins that evade the adaptive immune system (abbreviated as VPEs). Before developing models using each molecular descriptor tested, robust hyperparameter optimizations were performed on the training dataset, made up of 80% of the data, and subjected to a 10-fold cross-validation step. Cross-validation is a statistical technique used to assess the performance and generalizability of a predictive model. It involves dividing a dataset into multiple subsets, typically a training set and a validation set, multiple times to ensure robust model evaluation. This helps to mitigate the risk of overfitting and provides a more reliable estimate of how well the model will perform on new, unseen data [22, 23]. Subsequently, the optimized models were tested on the remaining 20% of the data (unseen data). The hyperparameters and their associated values were as follows, *n_estimators*: the number of trees in the forest. The possible values range from 100 to 1000, with increments of 100. *criterion*: The function to measure the quality of a split. The possible values are ‘gini’ (Gini impurity) and ‘entropy’ (information gain). *max_depth*: The maximum depth of the tree. The possible values are 5, 10, 15, 20, 25, 30 and None (unlimited depth). *min_samples_split*: The minimum number of samples required to split an internal node. The possible values are 2, 5 and 10. *min_samples_leaf*: The minimum number of samples required to be at a leaf node. The possible values are 1, 2 and 4. *max_features*: The number of features to consider when looking for the best split. The possible values are ‘sqrt’ (square root of the total number of features), ‘log2’ (log2 of the total number of features) and None (all features). *bootstrap*: Whether bootstrap samples are used when building trees. The possible values are True and False. *class_weight*: Weights associated with classes. The possible values are None (all classes have equal weight), ‘balanced’ (weights are inversely proportional to class frequencies) and ‘balanced_subsample’ (similar to ‘balanced’ but computed based on the bootstrap sample for every tree). *min_weight_fraction_leaf*: The minimum weighted fraction of the sum total of weights required to be at a leaf node. The possible values are 0.0, 0.1 and 0.2. *max_leaf_nodes*: The maximum number of leaf nodes in the tree. The possible values are None (unlimited leaf nodes), 5, 10, 20 and 50. *ccp_alpha*: Complexity parameter used for Minimal Cost-Complexity Pruning. The possible values are 0.0, 0.1 and 0.2. The scikit-learn machine learning library (https://scikit-learn.org/) was used for the entire workflow during the development of all predictive models. The following performance measures were evaluated for this binary classification problem:


(1) \begin{equation*} \mathrm{Sensitivity}\ \left(\mathrm{TP}\mathrm{R}\right)=\mathrm{TP}/\left(\mathrm{TP}+\mathrm{FN}\right) \end{equation*}

(2) \begin{equation*} \mathrm{Accuracy}\ \left(\mathrm{ACC}\right)=\mathrm{TP}+\mathrm{TN}/\left(\mathrm{TP}+\mathrm{FP}+\mathrm{FN}+\mathrm{TN}\right) \end{equation*}

(3) \begin{equation*} \mathrm{Precision}\ \left(\mathrm{PPV}\right)=\mathrm{TP}/\left(\mathrm{TP}+\mathrm{FP}\right) \end{equation*}

(4) \begin{equation*} \mathrm{F}1\ \mathrm{score}\ \left(\mathrm{F}1\right)=2\mathrm{TP}/\left(2\mathrm{TP}+\mathrm{FP}+\mathrm{FN}\right) \end{equation*}

(5) \begin{equation*} \mathrm{Specificity}\ \left(\mathrm{TNR}\right)=\mathrm{TN}/\left(\mathrm{FP}+\mathrm{TN}\right) \end{equation*}

(6) \begin{align*} k\left(\mathrm{kappa}\right)=2\ast \left(\mathrm{TP}\ast \mathrm{TN}-\mathrm{FN}\ast \mathrm{FP}\right))/\left(\mathrm{TP}+\mathrm{FP}\right)\ast \left(\mathrm{FP}+\mathrm{TN}\right)+\nonumber\\\left(\mathrm{TP}+\mathrm{FN}\right)\ast \left(\mathrm{FN}+\mathrm{TN}\right) \end{align*}

(7) \begin{equation*} \mathrm{MCC}=\left(\mathrm{TP}\right)\left(\mathrm{TN}\right)-\left(\mathrm{FP}\right)\left(\mathrm{FN}\right)/\sqrt{\left(\mathrm{TP}+\mathrm{FP}\right)\left(\mathrm{TP}+\mathrm{FN}\right)\left(\mathrm{TN}+\mathrm{FP}\right)\left(\mathrm{TN}+\mathrm{FN}\right)} \end{equation*}

with Matthew’s correlation coefficient (MCC), true positive (TP), false positive (FP), true negative (TN) and false negative (FN). Along with these performance measures, the receiver operating characteristic (ROC) curve was also evaluated at all stages of predictive model assessment. The ROC curve compares two operating characteristics (TPR and FPR), where TPR is the sensitivity mentioned earlier and FPR is the false positive rate defined as


(8) \begin{equation*} FPR= FP/\left( FP+ TN\right) \end{equation*}

**Figure 1:**
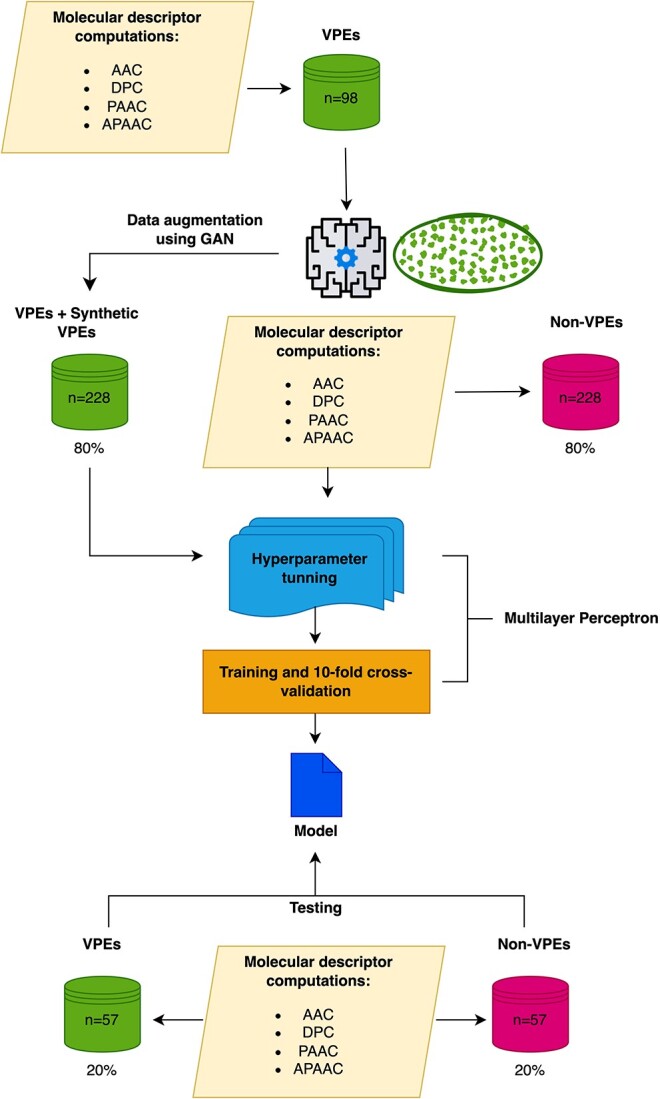
Workflow addressed in the present study. In the first stage, data were extracted from the UniProt platform. Next, the positive datasets were augmented using a GAN, based on four types of calculated molecular descriptors. Finally, the whole datasets (positive + negative) were divided and used to train and test predictive VPE models.

On the other hand, a web application named VirusHound-I was developed using the Python 3.11 programming language (https://www.python.org/). This web application scores the outputs with a probability ranging from 0 to 1. Figure 1 shows all the workflow implemented in this study (Figure 1).

## RESULTS

**Figure 2:**
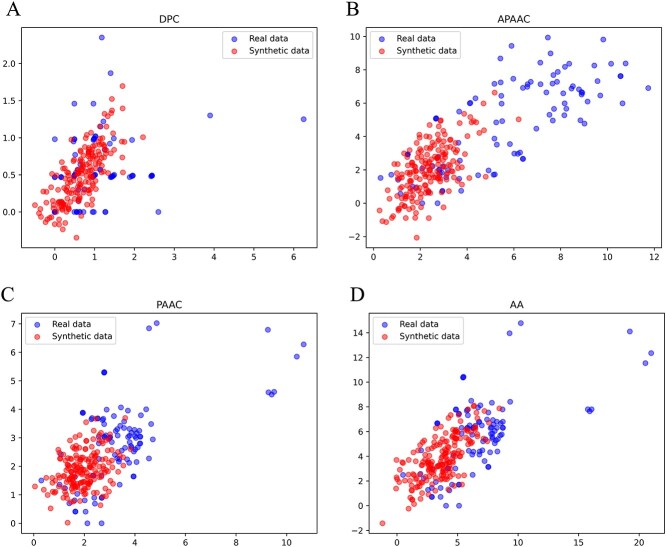
Plots of real values corresponding to each evaluated molecular descriptor and false values generated with the GAN.

**Figure 3:**
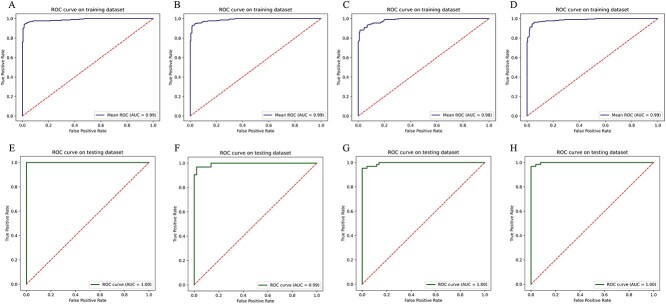
ROC curve for each molecular descriptor evaluated in the training and testing stages. AA (**A** and **E**), APAAC (**B** and **F**), PAAC (**C** and **G**) and DPC (**D** and **H**).

In this work, using a GAN-based strategy and starting from the four molecular descriptors evaluated, synthetic data were generated to augment the positive dataset. The plots of the molecular descriptor values for the synthetic data, when compared with the real data, showed that both exhibit a high similarity in all cases (Figure 2). Taking these results into account, datasets were created for the subsequent training and testing phases. The synthetic data was used to augment the positive datasets for VPE classification, by using RF (Figure 1). The 10-fold cross-validation on the training dataset showed that, in general, all evaluated descriptors allow obtaining predictive models with good performance according to the assessed metrics (Table 1 and Figure 3).

**Table 1:** Performance measures were obtained through 10-fold cross-validation on the training data, with the testing phase conducted using RF

MD/P	ACC	F1	PPV	TNR	TPR	*k*	MCC	AUC
AA/CVTr	0.942	0.939	0.966	0.970	0.914	0.885	0.886	0.986
AAC/Te	0.956	0.958	1.0^Te^	1.0^Te^	0.920	0.912	0.915	0.998
APAAC/CVTr	0.953^Tr^	0.951^Tr^	0.985	0.987	0.918^Tr^	0.907^Tr^	0.909^Tr^	0.989^Tr^
APAAC/Te	0.947	0.95	1.0^Te^	1.0^Te^	0.904	0.894	0.899	0.995
DPC/CVTr	0.947	0.943	0.994^Tr^	0.995^Tr^	0.896	0.894	0.898	0.989^Tr^
DPC/Te	0.964^Te^	0.967^Te^	1.0^Te^	1.0^Te^	0.936	0.929^Te^	0.931^Te^	1.0T^e^
PAAC/CVTr	0.929	0.926	0.943	0.948	0.909	0.859	0.859	0.984
PAAC/Te	0.964^Te^	0.967^Te^	0.983	0.980	0.952^Te^	0.929^Te^	0.929	0.995

The measures obtained from the 10-fold cross-validation represent the averaged values from each fold.

MD/P: molecular descriptor/phase, CVTr: 10-fold cross-validation phase on training data, Te: testing phase, AAC: amino acid composition, APAAC: amphiphilic pseudo amino acid composition, DPC: dipeptide composition, PAAC: pseudo amino acid composition.

On the other hand, good performance measures were also observed during the testing step, demonstrating the efficiency of the models in generalizing over new data (Table 1 and Figure 3). While all models presented excellent performance measures during the mentioned stages, we highlight the predictive model based on the DPC molecular descriptor because it showed the best performance measures on the test dataset, indicating better generalization over new data (Table 1 and Figure 3H). In this regard, this model was selected and incorporated into our web application VirusHound-I for VPE predictions. Considering the limited computational resources available to us to date, we limited the analysis to only 100 virus sequences per query. However, this number will gradually increase in the future as much as possible. The VirusHound-I tool is freely available at https://www.biochemintelli.com/VirusHound-I.

## DISCUSSION

Throughout evolution, pathogenic viruses of humans and animals have developed numerous strategies to camouflage themselves from the immune system [12, 24]. One of these strategies is the coding of proteins that evade the adaptive immune system, which is crucial for the successful replication of these pathogens in host cells [7, 25]. Therefore, identifying such viral proteins is crucial to developing vaccines and therapeutic drugs that can help combat these viruses.

In recent years, machine learning techniques have been key in the development of bioinformatics tools for studying virus proteins like the examples mentioned above [15–18], as well as others for the discovery of peptides with antiviral activity [26–34], which have experienced a recent explosion as a result of the lessons learned from the COVID-19 pandemic crisis. Discovering viral proteins that evade the immune system is a challenging task through both *in vivo* and *in vitro* experimental methods [35–37]. Firstly, in *in vivo* studies, which involve observing the interaction between the virus and the immune system within living organisms, numerous ethical and practical limitations arise. The use of animal models or even humans for such research raises ethical dilemmas concerning exposure to potentially harmful viruses [38]. In addition, viruses can evolve rapidly, further complicating the detection of proteins evading the immune system in some scenarios [2, 5, 35–37, 39–41]. On the other hand, *in vitro* studies conducted in controlled laboratory environments with cell cultures also present significant challenges in discovering these proteins. Viruses are inherently complex, and *in vitro* systems often oversimplify viral interactions, which could lead to inaccurate or incomplete results [42, 43]. Ultimately, *in vitro* methods often require sophisticated and costly techniques, limiting their applicability on a larger scale.

While it is true that there has been an increase in research in the field of machine learning applied to the study of pathogenic viruses in recent years, to date, there are no studies focused on the prediction of VPEs. Within the field of deep learning, GANs have been shown to be very useful in the development of bioinformatic predictive models [44]. For example, this type of neural network has been used as a data augmentation technique in the study of protein post-translational modifications [45], antiviral peptides [28] and protein solubility [46], among other cases reported in an excellent review by Wan *et al*. [44], showing excellent results. The results of our study regarding data augmentation using the proposed GAN (Figure 2) allowed us to obtain excellent models with the four molecular descriptors evaluated (Figure 3 and Table 1), corroborating the robustness of this technique to deal with few data. It is important to highlight that, although in this study there were cases where the evaluated molecular descriptors represented low (AAC = 20 features, APAAC = 50 features, APAA = 50 features) and high (DPC = 400 features) dimensionality, in all cases, good performance measures were obtained using synthetic data generated with the GAN. In fact, our study corresponds to what has been reported by Wan and Jones, who demonstrated that through a GAN-based approach, this type of neural network accurately learns the high-dimensional distributions of biophysical features based on protein sequences, as well as allowing the generation of high-quality synthetic protein feature samples that improve predictive model performance measures [47].

All the molecular descriptors in general allowed the development of predictive models of VPEs with good performance (Table 1 and Figure 3). However, we determined that models based on the molecular descriptors APAAC and DPC showed the best performances in predicting VPEs according to the metrics calculated during cross-validation and the testing stage (Table 1 and Figure 3). It is important to note that although both models performed well, they do exhibit differences. The APAAC-based model performed better in the training phase than the DPC-based model; however, the latter showed better performance on the independent test set. In this regard, we consider the DPC-based model to be a better choice for predicting VPEs, taking into account its better ability to generalize to new data. On the other hand, the computational resources needed to carry out predictions based on DPC are significantly lower than those required for APAAC. The utility of the DPC molecular descriptor has been widely demonstrated in many studies focused on the development of predictive models. For example, it has been used for the prediction of subcellular localization of eukaryotic proteins [48], antioxidant proteins [49], multiple subcellular localization of viral proteins [17], phage virion proteins [50], protein–protein interactions [51], thermophilic proteins [52], druggable proteins [53] and antifreeze proteins [54], among other studies. Consequently, considering the excellent performance of this molecular descriptor in our work, as well as the background of its successful use in the development of models based on machine learning, the DPC-based model was selected for the prediction of VPEs with the VirusHound-I tool.

Studying the proteins that allow viruses to evade the adaptive immune system is key to understanding how these pathogens infect host cells. Currently, there are some specific tools for studying viruses; however, none of them allow the prediction of VPEs. Therefore, we propose a machine learning-based tool called VirusHound-I for VPE predictions. VirusHound-I is a powerful tool based on a model developed with the DPC molecular descriptor, which showed good performance during the training and testing stages. The primary innovation of VirusHound-I is to expedite the large-scale discovery of VPEs, which would otherwise be unattainable using conventional *in vitro* and *in vivo* methods without significant resource and time expenditure. This tool can be employed as a preliminary step prior to laboratory experiments to explore and reduce the number of VPE candidates. We believe that VirusHound-I can be very useful in understanding the molecular mechanisms by which pathogenic viruses evade the adaptive immune response, as well as in discovering new therapeutic targets.

**Key Points**

- Pathogenic viruses have evolved diverse strategies to evade the adaptive immune system by encoding proteins that manipulate host cell mechanisms.
- Existing bioinformatics tools for virus research do not address the prediction of viral proteins that evade the adaptive immune system.
- The study introduces VirusHound-I, a novel machine learning-based tool using the Composition + Transition + Distribution molecular descriptor and generative antagonistic networks for data augmentation.
- VirusHound-I demonstrates high accuracy, precision and specificity in predicting viral proteins evading the host’s adaptive immune system, providing insights into the molecular mechanisms of pathogenic virus evasion and facilitating the discovery of potential therapeutic targets.

## Author Biographies

**Jorge F. Beltrán** is an expert in Bioinformatics and Data Science, focusing on Machine Learning and Deep Learning applied to biology and chemistry. He holds a PhD in Applied Cellular and Molecular Biology. Additionally, he has specialized training in Artificial Intelligence, excelling in the analysis and interpretation of large biological and chemical data sets. His primary fields of study are bioinformatics, artificial intelligence, and molecular and cellular biology, tackling scientific challenges with advanced data analysis techniques.

**Lisandra Herrera Belén** is an academic and researcher at the University of Santo Tomás in Temuco, Chile, specializing in bioinformatics, genetic engineering, and pharmaceutical chemistry. Her work focuses on the design of a chimeric (recombinant) enzyme using advanced bioinformatics tools, a project that merges aspects of biochemistry and pharmacology. Additionally, she has conducted research in the genetic modification of asparaginase, an enzyme from Escherichia coli, marking a significant advancement in the field of pharmaceutical chemistry and enzyme therapy.

**Jorge Farías Avendaño**, newly elected as the dean of the Faculty of Engineering and Sciences at the University of La Frontera, holds a distinguished academic and scientific profile. He is a biochemist from the Pontifical Catholic University of Valparaíso and holds a PhD in Biotechnology from the Federico Santa María Technical University and Pontifical Catholic University of Valparaíso. Dr. Farías is an active member of several national and international scientific societies, including the Chilean Society of Reproduction and Development, the Pharmacology Society of Chile, The American Society For Cell Biology, The American Physiology Society, the American Society of Andrology, and the American Society for Biochemistry and Molecular Biology.

**Mauricio Zamorano** is an Assistant Professor in the Department of Chemical Engineering at the University of La Frontera in Temuco, Chile. His primary research interest focuses on stem cell processing in three-dimensional (3D) environments, the design of medical devices, and clinical-scale bioreactors. Zamorano has a special interest in the production of bone, cartilaginous, and dental tissues on biodegradable scaffolds. In his research, he employs advanced techniques such as media perfusion, biophysical signaling, and the use of physiological levels of cytokines to optimize cell growth and differentiation in these environments. His work significantly contributes to the field of tissue engineering and regenerative medicine.

**Nicolás Lefin** is a graduate student at the University of La Frontera, Chile. In 2023, he graduated with honors in Chemical Civil Engineering from the same university, where he received the `Faculty Award' in recognition of his academic excellence and outstanding contributions. Currently, Nicolás is continuing his academic training as a master's student in Biotechnology Engineering at the University of La Frontera, in collaboration with University of São Paulo, Brasil. Specializing in the fields of bioprocessing, molecular biology, and protein engineering.

**Javiera Miranda** is a graduate student at the University of La Frontera, Chile. In 2023, he graduated with honors in Biotechnology Engineering from the same university, where he received the `Faculty Award' in recognition of his academic excellence and outstanding contributions. Currently, Javiera is continuing his academic training as a master's student in Biotechnology Engineering at the University of La Frontera, in collaboration with University of São Paulo, Brasil. Specializing in the fields of bioprocessing, molecular biology, and protein engineering.

**Fernanda Parraguez** is a master's student at the University of La Frontera, recently graduated with academic excellence from the Civil Engineering in Biotechnology program. She is currently continuing her studies in the Master of Science in Engineering program, specializing in Biotechnology at the University of La Frontera, focusing on water treatment, nanotechnology, and photocatalysis.
